# Acute and Early HIV Infection Among Key Populations in Rwanda: A Multisite Cross‐Sectional Study to Inform Targeted Testing and Case‐Finding, 2025

**DOI:** 10.1155/jotm/3534554

**Published:** 2026-11-15

**Authors:** Gallican Rwibasira, Jean Claude Kwizera, Steven Karera, Bruce Rwagitinywa, Everest Turatsinze, Alain Favina, Hackim Mugisha, Aflodis Kagaba, Gerard Kaberuka, Emmanuel Nkurunziza, Evans Mathebula, Tafadzwa Dzinamarira

**Affiliations:** ^1^ HIV, STI Viral Hepatitis and Other Viral Diseases Control Division Rwanda Biomedical Centre Kigali Rwanda rbc.gov.rw; ^2^ Swiss Tropical and Public Health Institute Allschwil Basel Switzerland swisstph.ch; ^3^ Department of Clinical Research Division of Clinical Epidemiology University of Basel Basel Switzerland unibas.ch; ^4^ Technical Department ICAP Kigali Rwanda; ^5^ Department of Epidemiology and Biostatistics School of Public Health College of Medicine and Health Sciences University of Rwanda Kigali Rwanda ur.ac.rw; ^6^ Technical Department Health Development Initiative Kigali Rwanda; ^7^ Infectious Diseases and Medical Diagnostics Department Abbott Rapid Diagnostics Johannesburg Gauteng South Africa; ^8^ School of Health Systems and Public Health Faculty of Health Sciences University of Pretoria Pretoria South Africa up.ac.za

**Keywords:** acute/early HIV infection, fourth-generation rapid test, high-risk populations, p24 antigen

## Abstract

**Background:**

Acute HIV infection is highly transmissible yet often missed by antibody‐only rapid tests. Rwanda adopted fourth‐generation antigen/antibody (Ag/Ab) tests in 2016, but routine p24 antigen documentation remains limited, limiting acute/early infection estimates. This study characterized acute/early HIV infection among key populations, including female sex workers (FSW), men who have sex with men (MSM), and people who inject drugs (PWID), to inform targeted testing and prevention interventions.

**Methods:**

We conducted a cross‐sectional study in 2025 across 25 health facilities in 12 Rwandan districts. Individuals aged ≥ 15 years with unknown HIV status from FSW, MSM, PWID, and adolescent girls and young women (AGYW) were recruited via snowball sampling. Structured RedCap questionnaires captured socio‐demographic, behavioral, and HIV testing history data. HIV testing followed the national serial algorithm using fourth‐generation Determine HIV Early Detect rapid tests; acute/early infection required p24 antigen positivity regardless of antibody status.

**Results:**

Among 1341 participants (median age 25 years, interquartile range [IQR]: 21–30), 864 (64%) were FSW, 177 (13%) were MSM, 58 (4.3%) were PWID, and 242 (18%) were AGYW; 66% had only primary education and 55% resided in rural areas. In the prior 4 weeks, 52% reported multiple sexual partners and 35.5% reported consistent condom use. One‐third (416, 32.8%) were aware of pre‐exposure prophylaxis (PrEP), and 34.7% (141/406) had ever used it. Overall HIV positivity was 5.0% (67/1341); acute/early HIV infection yield was 0.45% (6/1341), with two‐thirds of cases (4/6) among MSM, all from Kigali City. Half (3/6) of acute/early cases reported inconsistent condom use and none reported PrEP use.

**Conclusion:**

Acute and early HIV infections appear more common among MSM in Kigali, potentially associated with ongoing risk behaviors and low PrEP uptake, although this observation is based on a small number of cases. Priorities include strengthened acute HIV surveillance via fourth‐generation and self‐testing, rapid antiretroviral therapy (ART) initiation, and scaled‐up tailored prevention, especially PrEP.

## 1. Background

The global HIV/AIDS epidemic remains a significant public health challenge, with an estimated 40.8 million people living with HIV and 1.3 million new infections occurring in 2024 [1]. Despite a 40% reduction in new infections since 2010, high‐risk populations including female sex workers (FSW), men who have sex with men (MSM), people who inject drugs (PWID), and transgender women continue to bear a disproportionate burden of the epidemic [2, 3]. Globally, the median HIV prevalence among these high‐risk populations is substantially higher than the general adult prevalence of 0.7%, with MSM experiencing a prevalence of 7.6%, PWID at 7.1%, and FSW at 2.7% in countries reporting between 2020 and 2024 [4]. Notably, high‐risk populations and their partners accounted for 55% of all new HIV infections globally in 2022, highlighting the concentrated nature of transmission within these groups [1, 4]. The proportion of new infections among MSM increased from 11% in 2010 to 20% in 2022, while infections among PWID rose from 6.8% to 8.0% during the same period [4].

In Rwanda, substantial progress has been made in reducing HIV incidence and expanding access to antiretroviral therapy (ART), with the national adult HIV prevalence stabilizing at 3.0% among individuals aged 15–64 years according to the 2018–2019 Rwanda Population‐based HIV Impact Assessment (RPHIA) [5, 6]. However, the epidemic exhibits a mixed pattern demonstrating concentrated high burden among high‐risk populations. FSW face a particularly elevated risk, with HIV prevalence estimates at 35.2%, representing more than 10‐fold higher burden compared to the general population [7]. MSM have an estimated HIV prevalence of 5.8%, nearly twice the national average [8]. Adolescent girls and young women (AGYW) aged 15–24 years also experience disproportionate vulnerability, with prevalence rates three times higher than their male counterparts and increasing new infections in this demographic [5, 9].

Acute and early HIV infection (AEHI) represents a critical phase in the natural history of HIV transmission, characterized by rapid viral replication and extraordinarily high viral loads in blood and genital secretions [10, 11]. During the acute phase, which typically lasts 3‐4 weeks following HIV acquisition, plasma viral loads can exceed 1 million copies/mL, with peak concentrations occurring approximately 17 days after exposure in plasma and 30 days in semen. This substantial viral burden is associated with a markedly increased risk of HIV transmission, with the estimated hazard of transmission during acute infection being up to 26 times higher than during chronic infection [10]. Consequently, despite its brief duration, AEHI contributes disproportionately to onward sexual transmission of HIV, particularly in high‐risk populations where sexual networks facilitate rapid spread [11, 12]. The acute phase occurs during the “window period,” defined as the interval between HIV acquisition and the detection of antibodies by standard testing methods [13]. Traditional antibody‐only rapid diagnostic tests (RDTs) often fail to detect infection during this window, which can last 3–12 weeks, allowing highly infectious individuals to unknowingly transmit HIV [11, 14]. This diagnostic gap significantly undermines prevention and treatment strategies, including “Treat All” approaches, as individuals with acute infections may continue viral transmission before seroconversion [13].

The fourth‐generation HIV rapid diagnostic tests represent a significant advancement in early HIV detection by simultaneously detecting both HIV‐1/2 antibodies and HIV‐1 p24 antigen, a viral capsid protein present at high concentrations during early infection before antibody development. These combination antigen‐antibody assays can detect HIV infection as early as 14 days post‐exposure, with a median window period of 18 days, reducing the diagnostic gap by 4–7 days compared to third‐generation antibody‐only tests [15–17]. The detection of p24 antigen serves as a critical biomarker for identifying individuals in the acute phase of infection, enabling earlier treatment initiation and interruption of transmission chains, which are essential for epidemic control [15, 18]. Rwanda adopted fourth‐generation HIV testing within its national HIV testing algorithm, with the Alere Determine HIV‐1/2 Ag/Ab Combo test utilized as the first‐line screening assay in the national diagnostic algorithm [19].

Despite the incorporation of fourth‐generation tests into Rwanda’s national HIV testing strategy since 2016, a significant knowledge gap exists regarding the routine documentation and reporting of p24 antigen results. While recency testing has been implemented since 2018 to identify infections occurring within the preceding 12 months, this approach does not specifically isolate or characterize the acute phase of infection [20]. To date, no published data exist on individuals with acute HIV infection identified through p24 antigen detection in Rwanda, limiting the ability to estimate the true burden of AEHI among high‐risk populations. This documentation gap impedes accurate assessment of acute infections, hinders the design of targeted prevention interventions, and constrains evidence‐based policy development to prevent new transmissions. Systematic documentation of p24 antigen results would provide real‐time surveillance data on acute infections, particularly among high‐risk populations such as FSW, MSM, PWID, and AGYW, who account for a disproportionate share of new infections and would benefit most from early detection and immediate linkage to treatment and prevention services.

Therefore, we conducted this study to describe the epidemiological profile of AEHI among high‐risk populations in Rwanda, characterize the demographic and behavioral risk factors associated with AEHI, and evaluate the yield of p24 antigen detection using fourth‐generation rapid diagnostic tests. The findings will inform evidence‐based recommendations for strengthening early HIV detection strategies and guide targeted prevention efforts to reduce transmission among populations at highest risk.

## 2. Methods

### 2.1. Study Design and Period

We conducted a cross‐sectional study in 2025, to assess the epidemiological profile, behavioral risk factors, and yield of AEHI among high‐risk populations in Rwanda. This quantitative design enabled data collection at a single time point to estimate the prevalence of early and acute HIV infections and identify associated demographic and behavioral characteristics.

### 2.2. Study Setting and Sites

The study was conducted in 25 health facilities across 12 districts in Rwanda, selected based on an analysis of Health Management Information System (HMIS) data from 2022 to 2025. Districts were prioritized based on high HIV positivity rates (yield) among high‐risk groups, ensuring nationwide representation across all provinces including the City of Kigali. Within each selected district, one to two health facilities providing friendly services to high‐risk populations were identified to host the study. These facilities were chosen in collaboration with representatives of high‐risk groups through their community‐based organizations (CBOs). The selected districts included Musanze, Rubavu, Kamonyi, Rulindo, Kayonza, Nyamasheke, Gasabo, Kirehe, Nyarugenge, Kicukiro, Bugesera, and Muhanga, representing geographic diversity and areas with documented high HIV testing yield among high‐risk populations and AGYW.

### 2.3. Study Population

The study population comprised individuals aged 15 years and above from high‐risk populations with unknown HIV status. High‐risk populations included FSW, MSM, PWID, and AGYW. These groups were selected based on their disproportionately high HIV incidence rates and significant contribution to new transmissions in Rwanda.

Inclusion criteria required participants to be aged 15 years or older, capable of providing informed consent (or, for participants aged 15–17 years, assent together with consent from a parent or legal guardian), independently or with a witness for individuals with low literacy, self‐identifying as active members of a high‐risk group (FSW, MSM, PWID, or AGYW) and being referred by a trained peer educator or key informant. Exclusion criteria included individuals already known to be living with HIV and currently on ART, those presenting with acute medical instability requiring immediate critical care, and unwillingness to provide informed consent.

### 2.4. Sampling and Recruitment

A snowball sampling approach was employed to recruit participants from high‐risk populations, a method particularly effective for reaching hidden or hard‐to‐reach populations where no comprehensive sampling frame exists. Initial participants, or “seeds,” were identified through collaboration with established community‐based organizations, specifically Health Development Initiative (HDI) Rwanda and other local non‐governmental organizations actively working with these high‐risk groups. Trained peer educators and key informants, who possess in‐depth knowledge of and access to their respective communities, facilitated the recruitment of additional eligible participants from their networks. The recruitment process continued iteratively, with each participant potentially referring others from their social or professional networks, until the desired sample size was achieved. Throughout the recruitment process, participant safety, confidentiality, and voluntary participation were maintained as paramount principles.

### 2.5. Data Collection

Data were collected using structured questionnaires administered via Research Electronic Data Capture (REDCap), a secure web‐based platform designed for electronic data capture in research studies. The questionnaires captured socio‐demographic characteristics including age, sex, education level, marital status, occupation, district of residence, and urban versus rural residency. Sexual behavioral data included number of sexual partners in the past 4 weeks, condom use and ability to negotiate condom use patterns, awareness and use of pre‐exposure prophylaxis (PrEP), and HIV testing history.

### 2.6. HIV Testing Procedures

HIV testing was conducted following the Rwanda National HIV Testing Algorithm using fourth‐generation rapid diagnostic tests capable of simultaneously detecting HIV‐1/2 antibodies and HIV‐1 p24 antigen. The national serial testing algorithm employed the Alere Determine HIV‐1/2 Ag/Ab Combo test as the first‐line screening assay, followed by the HIV 1/2 Stat‐Pak (Chembio Diagnostic Systems) as the confirmatory test. A non‐reactive result on the first test classified the individual as HIV negative. Reactive results on both the first and second tests classified the individual as HIV positive. Specimens with a reactive first test followed by a non‐reactive second test were considered inconclusive and excluded from analysis.

AEHI was defined by the presence of p24 antigen on the fourth‐generation rapid test strip, indicated by a visible line at the p24 antigen detection zone. This definition identifies individuals in the acute phase of infection, during which high viral replication occurs before full seroconversion. All HIV testing was performed by certified healthcare providers who had undergone specific training in HIV testing and counseling, following established standard operating procedures to ensure accuracy and reliability. Continuous quality assurance measures were implemented, including internal quality control, external quality assessment, and ongoing supervision. All individuals who tested HIV positive received post‐test counseling and were immediately linked to HIV care and treatment services according to national guidelines.

### 2.7. Study Outcomes

The primary outcome was the overall HIV positivity rate, calculated as the proportion of participants testing HIV positive among all tested individuals. The secondary outcome was the yield of AEHI, defined as the proportion of participants with p24 antigen‐positive results among all tested individuals and among all HIV‐positive cases. Subgroup analyses examined HIV positivity and AEHI yield stratified by population type (FSW, MSM, PWID, AGYW), age groups, sex, geographic location (including Kigali City vs. other provinces, and urban versus rural residence), education level, sexual behavioral characteristics (number of sexual partners, condom use consistency), and PrEP awareness and utilization. Additional outcomes included the demographic and behavioral profiles of individuals identified with AEHI.

### 2.8. Data Management and Statistical Analysis

Data management was overseen by a dedicated data manager who conducted daily monitoring for completeness, consistency, and identification of missing variables. All collected data were stored securely within the REDCap system, which employs advanced security measures including password protection and role‐based access controls to protect sensitive health information. Regular data quality checks were performed, including verification against source documents, logical consistency checks, and prompt resolution of discrepancies.

Statistical analyses were performed using R statistical software, version 4.5.1 (R Foundation for Statistical Computing, Vienna, Austria, 2025) with the following packages: dplyr for data manipulation, gtsummary and flextable for table generation, ggplot2 for data visualization, and binom for confidence interval (CI) calculations. Descriptive statistics were used to characterize the study population and estimate HIV positivity and AEHI prevalence. Categorical variables were summarized as frequencies and percentages, while continuous variables were presented as medians with interquartile ranges (IQR).

Prevalence estimates were calculated with 95% CI using the Wilson score method for binomial proportions, which provides better coverage properties than the normal approximation, particularly for small samples or extreme proportions. Subgroup analyses compared HIV positivity and AEHI prevalence across different demographic and behavioral categories using Fisher’s exact test for categorical variables with small sample sizes and chi‐square tests for larger samples where test assumptions were met. Statistical significance was defined as a two‐sided *p* value less than 0.05.

### 2.9. Ethical Considerations

The study protocol received ethical clearance from the Institutional Review Board (IRB) of the College of Medicine and Health Sciences (CMHS), University of Rwanda (approval No. 824/CMHS IRB/2025). Additionally, a collaboration letter was obtained from the Rwanda Biomedical Centre to secure access to the health facilities required for the study. All study procedures were conducted in strict accordance with national ethical guidelines and international research ethics standards.

Comprehensive informed consent was obtained from all participants prior to enrollment. The consent process ensured that participants clearly understood the study objectives, procedures, potential risks and benefits, their rights, and measures to protect confidentiality. Participation was entirely voluntary and free from any form of coercion, and participants were informed of their right to withdraw from the study at any time without penalty or any effect on their access to healthcare services. For participants aged 15–17 years, verbal assent was obtained following an assessment of understanding by a trained counselor, while written informed consent was obtained from a parent or legal guardian, in accordance with Rwanda’s national HIV testing guidelines and ethical requirements for research involving minors. All information was provided in clear, accessible language (Kinyarwanda, English, or French), with interpreters engaged when necessary to address language barriers.

Confidentiality of all participant information was meticulously maintained throughout the study. All client files and records were stored securely with restricted access limited to authorized study personnel who had been trained on confidentiality protocols. Data were de‐identified using unique participant codes to remove direct personal identifiers before analysis and dissemination. Participant information was shared only when explicit written consent for its release had been obtained. The physical infrastructure used for service delivery was designed to facilitate private dialog and uphold confidentiality during all interactions, including HIV testing, counseling, and data collection procedures.

## 3. Results

### 3.1. Sociodemographic Characteristics of Study Participants

A total of 1341 participants were enrolled, and the study population comprised 864 (64.4%) FSW, 177 (13.2%) MSM, 58 (4.3%) PWID, and 242 (18.0%) AGYW. The median age of participants was 25 years (IQR: 21–30 years).

The majority of participants were female (1111 [82.8%]), which reflects the predominance of FSW and AGYW in the study population. Educational attainment was generally low, with 75.2% being illiterate or completing only primary education. Most participants reported being single (86.7%) and resided in rural areas (54.5%). Educational level varied by population type, with MSM having higher proportions with secondary or higher education (48.0%) compared to other groups (Table 1).

**TABLE 1:** Sociodemographic characteristics of study participants by population type.

Characteristic	Overall *N* = 1,341^1^	Key population groups			
		FSW *N* = 864^1^	AGYW *N* = 242^1^	MSM *N* = 177^1^	PWID *N* = 58^1^
Age (years)	25 (21.0–30)	26 (22.0–31)	20 (19.0–22)	27 (23.0–31)	30 (25.0–36)
Age group					
15–19 years	185 (13.8)	85 (9.8)	86 (35.5)	11 (6.2)	3 (5.2)
20–24 years	448 (33.4)	233 (27.0)	156 (64.5)	48 (27.1)	11 (19.0)
25–29 years	362 (27.0)	288 (33.3)	0 (0.0)	62 (35.0)	12 (20.7)
30–34 years	159 (11.9)	114 (13.2)	0 (0.0)	29 (16.4)	16 (27.6)
35+ years	187 (13.9)	144 (16.7)	0 (0.0)	27 (15.3)	16 (27.6)
Sex					
Female	1111 (82.8)	864 (100.0)	242 (100.0)	0 (0.0)	5 (8.6)
Male	230 (17.2)	0 (0.0)	0 (0.0)	177 (100.0)	53 (91.4)
Education level					
None	120 (9.0)	87 (10.1)	20 (8.3)	6 (3.5)	7 (12.5)
Primary	881 (66.2)	607 (70.4)	163 (67.6)	84 (48.6)	27 (48.2)
Secondary or higher	331 (24.8)	168 (19.5)	58 (24.1)	83 (48.0)	22 (39.3)
Marital status					
Single	1158 (86.7)	742 (86.4)	225 (93.4)	141 (79.7)	50 (86.2)
Married/cohabiting	53 (4.0)	18 (2.1)	11 (4.6)	18 (10.2)	6 (10.3)
Separated/divorced/widowed	124 (9.3)	99 (11.5)	5 (2.1)	18 (10.2)	2 (3.4)
Residence					
Rural	731 (54.5)	465 (53.8)	148 (61.2)	84 (47.5)	34 (58.6)
Urban	610 (45.5)	399 (46.2)	94 (38.8)	93 (52.5)	24 (41.4)

*Note:* MSM = men who have sex with men. AGYW included in the study were aged 15–24 years; therefore, the age categories of 25 years and above had zero observations.

Abbreviations: AGYW, adolescent girls and young women; FSW, female sex workers; PWID, people who inject drugs.

^1^Median (IQR); *n* (%).

### 3.2. HIV Testing Outcomes

Of the 1341 participants tested using fourth‐generation HIV‐1/2 antibody/p24 antigen rapid diagnostic tests, 67 tested positive for HIV, yielding an overall HIV positivity rate of 5.0% (95% CI: 4.0%–6.3%). Among the HIV‐positive cases, 6 tested positive for p24 antigen, indicating AEHI. The overall AEHI prevalence was 0.45% (95% CI: 0.21%–0.97%) of all participants tested, and AEHI accounted for 9.0% (95% CI: 4.2%–18.2%) of all HIV‐positive cases (Table 2).

**TABLE 2:** HIV testing outcomes by population type.

Population	HIV positive *n*/*N* (%)	95% CI	AEHI (p24+) *n*/*N* (%)	95% CI	AEHI among HIV + *n*/*N* (%)	95% CI
Overall						
(*N* = 1341)	67/1341 (5.0)	4.0–6.3	6/1341 (0.45)	0.21–0.97	6/67 (9.0)	4.2–18.2
FSW						
(*N* = 864)	41/864 (4.7)	3.5–6.4	1/864 (0.12)	0.02–0.65	1/41 (2.4)	0.4–12.6
MSM						
(*N* = 177)	13/177 (7.3)	4.3–12.2	4/177 (2.26)	0.88–5.67	4/13 (30.8)	12.7–57.6
PWID						
(*N* = 58)	4/58 (6.9)	2.7–16.4	0/58 (0.00)	0.00–6.21	0/4 (0.0)	0.0–49.0
AGYW						
(*N* = 242)	9/242 (3.7)	2.0–6.9	1/242 (0.41)	0.07–2.30	1/9 (11.1)	2.0–43.5

*Note:* Fisher’s exact test (HIV positive by population): *p* = 0.30. AEHI = Acute and Early HIV Infection; MSM = men who have sex with men.

Abbreviations: AGYW, adolescent girls and young women; FSW, female sex workers; PWID, people who inject drugs.

HIV positivity showed significant variation across age groups (*χ* ^2^ = 21.282, *df* = 4, *p* < 0.001) (Figure 1). The highest HIV positivity was observed among participants aged 30 years and older, with those aged 35 years and above at 10.2% (19/187) and those aged 30–34 years at 6.3% (10/159). No significant difference in HIV prevalence was observed across key population groups (Fisher’s exact test: *p* = 0.30).

**FIGURE 1:**
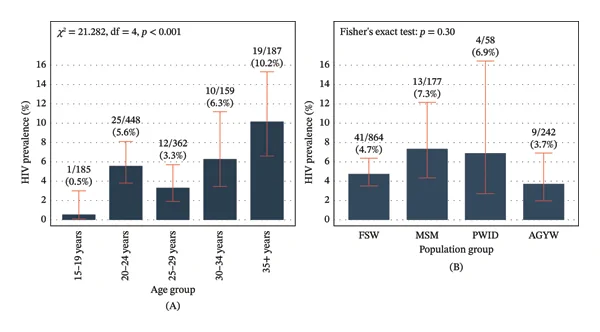
HIV positivity by age group (A) and by key population group (B) among 1341 study participants. Bars represent HIV prevalence (%); labels above each bar indicate the number of HIV‐positive participants over the number tested (*n*/N) and the percentage positive. Red error bars represent 95% CI calculated using the Wilson score method. Differences in (A) were assessed using a chi‐square test; differences in (B) were assessed using Fisher’s exact test, appropriate given small cell counts in some population groups. Abbreviations: FSW, female sex workers; MSM, men who have sex with men; PWID, people who inject drugs; AGYW, adolescent girls and young women.

### 3.3. Acute and Early HIV Infection Characteristics

Four of the six cases (66.7%) were identified among MSM, representing 2.26% of all MSM tested and 30.77% of HIV‐positive MSM, compared with one case each among FSW (0.12% of FSW tested, 2.44% of HIV‐positive FSW) and AGYW (0.41% of AGYW tested, 11.11% of HIV‐positive AGYW), and no AEHI cases among PWID (Table 2).

Geographically, four of the six AEHI cases (66.7%) were from Kigali City, specifically from Nyarugenge (3 cases) and Gasabo (1 case) districts, representing 1.28% of all participants tested in Kigali City. The remaining two cases were from Kayonza and Bugesera districts (0.19% of participants from other districts). This geographic concentration in the capital city suggests potential urban transmission hotspots and highlights the importance of enhanced surveillance in urban centers.

Regarding risk behavior profiles among AEHI cases, half of the AEHI cases (3/6, 50%) reported inconsistent condom use (sometimes or never), while the other half (3/6, 50%) reported consistent condom use. Regarding PrEP, while four AEHI cases (66.7%) were aware of PrEP, notably, none had ever taken PrEP despite being in high‐risk populations. All six AEHI cases (100%) reported having been previously tested for HIV with negative results.

### 3.4. Sexual Behavior and HIV Prevention Practices

More than half (51.7%, 692/1339) reported having 10 or more sexual partners in the 4 weeks preceding the survey, with only 35.5% (476/1340) of participants reporting consistent condom use during sexual intercourse, while the majority (64.5%, 864/1340) reported inconsistent use (sometimes or never). Notably, HIV‐positive participants were significantly less likely to report consistent condom use compared to HIV‐negative participants (19.4% vs. 36.4%, *p* = 0.004). Awareness and utilization of HIV prevention services were suboptimal with only 32.8% (416/1269) of participants had heard of PrEP, with nearly two‐thirds (67.2%) being unaware of this prevention method. Among those aware of PrEP, uptake was low, with only 34.7% (141/406) having ever taken it (Table 3).

**TABLE 3:** Comparison of sexual behavior and HIV prevention characteristics by HIV status.

Characteristic	Overall (*N* = 1341)	HIV negative *N* = 1274	HIV positive *N* = 67	*p* value^1^
Number of sexual partners (last 4 weeks)				0.42
None	44 (3.3%)	43 (3.4%)	1 (1.5%)	
1–4 partners	362 (27.0%)	338 (26.6%)	24 (35.8%)	
5–9 partners	241 (18.0%)	230 (18.1%)	11 (16.4%)	
10+ partners	692 (51.7%)	661 (52.0%)	31 (46.3%)	
Condom use				0.004
Consistent	476 (35.5%)	463 (36.4%)	13 (19.4%)	
Inconsistent	864 (64.5%)	810 (63.6%)	54 (80.6%)	
Ability to negotiate condom use (past month)				0.004
Never	205 (18.8%)	186 (17.9%)	19 (38.0%)	
Sometimes	724 (66.3%)	699 (67.1%)	25 (50.0%)	
Frequently	163 (14.9%)	157 (15.1%)	6 (12.0%)	
Ever tested for HIV				0.03
Yes	1175 (87.8%)	1122 (88.2%)	53 (79.1%)	
No	164 (12.2%)	150 (11.8%)	14 (20.9%)	
Heard of PrEP				> 0.99
Yes	416 (32.8%)	396 (32.8%)	20 (31.7%)	
No	853 (67.2%)	810 (67.2%)	43 (68.3%)	
Ever taken PrEP				0.47
Yes	141 (34.7%)	136 (35.2%)	5 (25.0%)	
No	265 (65.3%)	250 (64.8%)	15 (75.0%)	

*Note:* PrEP = pre‐exposure prophylaxis.

^1^Fisher exact test.

## 4. Discussion

This cross‐sectional study, conducted in 25 health facilities across 12 districts of Rwanda, provides the first documented evidence of AEHI prevalence among high‐risk populations using p24 antigen detection from fourth‐generation rapid diagnostic tests. Our findings reveal an overall HIV positivity rate of 5.0% (95% CI: 4.0%–6.3%) and an AEHI prevalence of 0.45% (95% CI: 0.21–0.97), representing 9.0% of all HIV‐positive cases. Notably, MSM accounted for 66.7% (4/6) of AEHI cases, and none of the individuals with AEHI had used PrEP despite awareness of this prevention method. These findings highlight critical gaps in early HIV detection and prevention uptake among populations at highest risk for HIV transmission.

The observed HIV positivity rate of 5.0% among high‐risk populations in our study is consistent with Rwanda’s concentrated epidemic pattern and aligns with previous surveillance data. While lower than the 35.2% prevalence reported among FSW in earlier Rwandan studies [7], our findings reflect a broader spectrum of high‐risk populations including MSM, PWID, and AGYW. The HIV prevalence among MSM (7.34%, 13/177) in our study is higher than the 5.8% national estimate [8] but lower than the pooled prevalence of 11.3% among MSM in Sub‐Saharan Africa [21]. Among FSW, our observed prevalence of 4.75% (41/864) was substantially lower than both the recent Rwandan estimate of 35.2% (9) and the 2024 pooled Eastern and Southern Africa prevalence of 36% [22], potentially reflecting geographic heterogeneity, temporal trends in prevention interventions, or sampling differences between studies. Across Sub‐Saharan Africa, HIV prevalence among FSW remains elevated at approximately 30 times the risk of other adult women, while MSM face 28 times the risk of other adult men [23]. The relatively moderate HIV prevalence in our study may also reflect successful prevention and treatment scale‐up in Rwanda over the past decade, although sustained vigilance among high‐risk populations remains essential.

The documented AEHI prevalence of 0.45% among all participants and 9.0% among HIV‐positive individuals represents a critical contribution to understanding the acute infection burden in Rwanda. To our knowledge, this is the first published estimate of AEHI prevalence among high‐risk populations in Rwanda using systematic p24 antigen documentation. Previous studies in Eastern and Southern Africa have estimated acute HIV infection prevalence between 1% and 3% of all tested individuals [24], suggesting our findings fall within the lower range of regional estimates. The proportion of AEHI among HIV‐positive cases is of substantial public health significance, as acute infection is characterized by extraordinarily high viral loads and higher transmission risk than during chronic infection [10]. Mathematical modeling studies have estimated that 11%–20% of all new HIV infections are transmitted by individuals in the acute phase, despite this phase lasting only 3–4 weeks [25, 26], while more than 50% can be transmitted by someone unaware of HIV status [25]. Therefore, although based on a small number of cases, the identification of six AEHI cases in our study may indicate individuals within active transmission chains, underscoring the potential value of their early detection for epidemic control.

The concentration of AEHI among MSM in our study, with 66.7% of cases occurring in this population despite MSM comprising only 13.2% of participants, warrants particular attention, although this finding is based on a small absolute number of cases (*n* = 6) and should be interpreted with caution. MSM accounted for four of the six AEHI cases, representing 30.77% of all HIV‐positive MSM. This concentration aligns with global evidence that MSM experience significantly elevated HIV acquisition risk, with biological, behavioral, and structural vulnerabilities contributing to this disparity [23, 27]. Condomless receptive anal intercourse, multiple sexual partners, and high rates of sexually transmitted infections (STIs) are well‐documented risk factors for acute HIV infection among MSM [28]. The efficiency of rectal mucosa in facilitating HIV transmission, combined with concentrated sexual networks within MSM communities, creates conditions for rapid viral dissemination during acute infection [29]. In our study, half of the AEHI cases reported inconsistent condom use, highlighting ongoing behavioral risk. The geographic clustering of AEHI cases in Kigali City (66.7%) further suggests urban transmission hotspots, consistent with Kigali’s documented higher HIV incidence rates [5] and the concentration of MSM networks in urban settings.

The implementation of fourth‐generation HIV testing using p24 antigen detection holds significant promise for public health impact, yet our findings underscore the critical gap in routine documentation of p24 results in Rwanda’s HIV program. Fourth‐generation tests can detect HIV infection as early as 14–18 days post‐exposure, reducing the diagnostic window period by 4–7 days compared to third‐generation antibody‐only tests [15, 16, 30]. This earlier detection is crucial for interrupting transmission chains during the highly infectious acute phase and facilitating immediate linkage to ART. A mathematical model estimated that early ART initiation during acute infection can reduce onward transmission by up to 88.7% in the first year of infection [31]. However, the public health benefits of fourth‐generation testing can only be realized if p24 antigen results are systematically documented, reported, and acted upon through targeted prevention interventions. The current lack of routine p24 documentation in Rwanda represents a missed surveillance opportunity that limits the ability to monitor acute infection trends, allocate resources to transmission hotspots, and design evidence‐based prevention strategies for populations at highest risk.

A particularly concerning finding in our study is the complete absence of PrEP use among all six individuals identified with AEHI, despite four of them being aware of PrEP’s existence. This represents a critical missed prevention opportunity, as PrEP has demonstrated high effectiveness (> 90%) in preventing HIV acquisition when taken consistently [32, 33]. Overall PrEP awareness in our study was low (32.8%), and uptake among those who were aware was also low (34.7%), consistent with challenges in PrEP implementation across Sub‐Saharan Africa. While a recent Rwandan study among high‐risk populations in Kigali City reported relatively high PrEP retention rates of 77% for FSW and 82% for MSM at 12 months among initiators [34], our findings suggest that PrEP initiation remains a significant barrier, with low HIV risk perception identified as a predictor of poor retention. Barriers to PrEP uptake in Africa include stigma, side effects concerns, logistical challenges, partner consultation requirements, and healthcare provider attitudes [35, 36]. The fact that individuals identified with AEHI had not initiated PrEP underscores the need for enhanced community mobilization, peer‐led interventions, and healthcare provider training to improve PrEP coverage among populations at substantial HIV risk.

The finding that HIV‐positive participants were significantly less likely to use condoms consistently compared to HIV‐negative participants is concerning and aligns with broader patterns observed in Sub‐Saharan Africa. A systematic review and meta‐analysis reported a pooled prevalence of consistent condom use among sexually active HIV‐positive individuals of 42.52% (95% CI: 20.29%–64.74%) across the region [37]. The low rate of consistent condom use among HIV‐positive individuals in our study highlights ongoing transmission risk. Interventions focusing on couples’ HIV testing, disclosure support, and education about the continued importance of condoms for dual protection against transmission and reinfection are essential.

Our study has several notable strengths. First, the large sample size of 1341 participants across diverse geographic areas and multiple key population groups provides robust prevalence estimates. Second, the multi‐site design encompassing 25 health facilities in 12 districts enhances geographic representativeness and captures heterogeneity in HIV burden across Rwanda. Third, the use of fourth‐generation HIV rapid diagnostic tests with systematic p24 antigen documentation represents a methodological advancement over previous studies that relied solely on antibody detection. Fourth, the inclusion of behavioral and prevention service utilization data enables a comprehensive characterization of risk profiles and prevention gaps among individuals with AEHI. Finally, the study addresses a critical knowledge gap regarding acute HIV infection in Rwanda, where no previous data on p24‐documented AEHI prevalence among high‐risk populations have been published.

However, several limitations must be acknowledged. First, the cross‐sectional design precludes causal inference regarding the temporal relationship between risk behaviors and HIV acquisition and provides only a single time point estimate of prevalence without capturing incidence trends. Second, the use of snowball sampling, while appropriate for hard‐to‐reach populations, introduces inherent selection bias favoring individuals with larger social networks and those more willing to participate, potentially limiting generalizability to the broader key population. Third, our definition of AEHI relied solely on p24 antigen positivity without viral load confirmation or HIV RNA testing, which would have provided more precise staging of infection and identification of pre‐seroconversion acute infection. Fourth, the small number of AEHI cases identified (*n* = 6) limits the precision and generalizability of subgroup estimates, as reflected in the wide CI around population‐ and geography‐specific findings; these results should therefore be interpreted as preliminary and hypothesis‐generating rather than definitive. Finally, self‐reported behavioral data are subject to recall bias and social desirability bias, potentially leading to over‐reporting of condom use and under‐reporting of risk behaviors.

Based on our findings, we recommend several actions for policy and practice. First, Rwanda’s HIV program should implement routine documentation and reporting of p24 antigen results from fourth‐generation tests to enable real‐time surveillance of acute infections and guide resource allocation to transmission hotspots. Second, given that most AEHI cases in this study were identified among MSM in Kigali City, targeted AEHI screening and immediate treatment linkage should be prioritized for MSM in urban centers, although this pattern is based on a small number of cases and warrants confirmation in larger studies. Third, comprehensive PrEP scale‐up is urgently needed, with community‐based delivery models, peer navigation, and demand creation strategies tailored to each key population’s specific barriers and preferences. Fourth, integration of frequent STI screening with HIV testing services could enhance AEHI detection, given the association between STIs and acute HIV infection among MSM reported in prior literature. Fifth, enhanced condom promotion and distribution, coupled with partner notification and couples’ testing services, should be strengthened among HIV‐positive individuals and their partners. Finally, longitudinal cohort studies with viral load confirmation and larger AEHI case numbers would provide more robust incidence estimates and enable evaluation of interventions to reduce acute HIV transmission.

## Author Contributions

Gallican Rwibasira: conceptualization, methodology, and writing–original draft. Jean Claude Kwizera: data collection and writing–review and editing. Steven Karera: data collection and writing–review and editing. Bruce Rwagitinywa: data collection and writing–review and editing. Everest Turatsinze: data collection and writing–review and editing. Alain Favina: data analysis and writing–review and editing. Hackim Mugisha: data analysis and writing–review and editing. Aflodis Kagaba: data analysis and writing–review and editing. Gerard Kaberuka: data analysis and writing–review and editing. Emmanuel Nkurunziza: supervision and writing–review and editing. Tafadzwa Dzinamarira: supervision and writing–review and editing.

## Funding

This study was supported by Abbott through HDI under Grant No. 16125212.

## Disclosure

The funder had no role in the study design, data collection, data analysis, interpretation of the findings, manuscript preparation, or the decision to publish.

## Conflicts of Interest

Evans Mathebula is employed by Abbott Rapid Diagnostics. The other authors declare no conflicts of interest.

## Data Availability

The data supporting the findings of this study are contained within the manuscript. Additional data are available from the corresponding author upon reasonable request.
